# Global landscape of community pharmacy services remuneration: a narrative synthesis of the literature

**DOI:** 10.1186/s40545-023-00626-0

**Published:** 2023-10-09

**Authors:** Rabia Hussain, Zaheer-Ud-Din Babar

**Affiliations:** 1 School of Pharmaceutical Sciences, Universiti Sains Malaysia, 11800 Penang, Malaysia; 2 University of Huddersfield, Queensgate, Huddersfield, HD1 3DH UK

**Keywords:** Community pharmacy, Remuneration, Reimbursement, Fee for service, Capitation, Cognitive pharmaceutical services

## Abstract

Community pharmacists form a vital part of the health system all around the globe. Pharmacy remuneration models are aimed to ensure that pharmacies are sustained, and pharmacists could provide cost-effective services to the patients. This review summarizes the pharmacy services remuneration systems from different parts of the globe. Some countries have well-established reimbursement systems that recognize and compensate community pharmacies for their services, others are in the process of expanding the scope of reimbursable services. It further concludes by highlighting the ongoing efforts to incorporate pharmacist-provided services into reimbursement schemes and the need for standardized and consistent approaches to pharmacy remuneration globally.

## Background

Over the decades, the profession of pharmacy has gone through substantial changes. From being a profession which merely focused on supply and distribution of medicines, the role of pharmacist has evolved over the time and became more focused towards patient care [1]. After physicians and nurses, pharmacists are the largest healthcare professionals workforce around the globe [2]. Moreover, community pharmacists are most accessible healthcare professionals and the way they provide services could impact on patient health outcomes [3–5]. The professional pharmacy services include medicine use reviews, medication adherence programs, screening and monitoring, etc. These services are referred to as extended pharmacy services or cognitive pharmaceutical services [6]. Cognitive pharmaceutical service is a parallel term used to describe the variety of pharmacy services and interventions developed to optimize pharmacotherapy through effective interaction with patients and healthcare professionals [7]. A cognitive pharmaceutical service is referred to any activity in which the pharmacists utilize their professional knowledge and expertise to promote safe and effective drug therapy [6].The studies have indicated that such services provided by the community pharmacists have a favorable impact on the healthcare system [8]. A review in United States based community pharmacies have concluded that pharmacist led interventions in the case of patients with diverse conditions such as diabetes, cardiovascular, hypertension and smoking have promoted positive outcomes [9–11]. Similarly, clinical medication reviews by community pharmacists have helped in identifying and sorting drug related problems including inappropriate prescribing, polypharmacy, and improved medication adherence [12–14].

Since the last two decades, community pharmacists have started establishing pharmacy services which are personalized in terms of healthcare delivery [15]. The personalized healthcare delivery allows the community pharmacists to assess and monitor the medicine use and patient’s condition to improve health-related outcomes [16, 17].

Many countries have implemented the professional pharmacy services, which are often subsidized by national healthcare system [15]. These services aim to optimize medication therapy and improve patient outcomes by providing specialized expertise in medication management. The financing of pharmacy services refers to the mechanisms and sources of funding that support the provision of these services within the healthcare system. The financing arrangements can vary between countries and healthcare systems. A report by International Pharmaceutical Federation (FIP) has stated that the most remunerated services include compounding of prescription medicines, medication reviews and vaccination at community pharmacies. Only in 13 countries or territories, pharmacy services are remunerated which involves improving the use of medicines. The remuneration is for those services which include clinical decision-making, treatment initiation, continuation, and modification. In the report, a total of 65 respondents stated that third-party remuneration exists for pharmacists. Out of 65, a total of 37 (57%) indicated that there is a single third-party payer, while 28 (43%) mentioned having multiple contractual agreements. It is being stated that having multiple agreements may allow pharmacists to have greater flexibility. However, these agreements are complex in nature and pharmacists need more administrative support in this regard [18]. As pharmacy remuneration only exists in a small number of countries, therefore the aim of this narrative review is to present most common models of reimbursement for pharmacy services as well as to provide a comprehensive overview of global reimbursement system of community pharmacy services.

### Community pharmacy remuneration and reimbursement

The expansion and funding of cognitive pharmaceutical services is happening globally, and many countries have opted for different models of reimbursement for different pharmaceutical services [19]. Pharmacy remuneration model, which is enforced by law, is known as statutory and the one which is determined through a legal document is known as contractual remuneration model. The remuneration in community pharmacy can be either statutory, contractual or combination of both. Moreover, community pharmacy services remuneration needs to consider the activities and services contributed by a certain healthcare system, value, and cost of delivery of services. The remuneration model should be designed in a way that optimal health outcomes can be achieved through the delivery of pharmaceutical products and services. Therefore, several components contribute towards the delivery of such services.

In most countries, pharmacy remuneration is concentrated on dispensing, which could be seen as margin, maximum reimbursement price or dispensing fee. The dispensing fee is remunerated in many ways, including prescription fee, fee per pack, per visit to a community pharmacy [20]. The other component of remuneration of community pharmacy services is regarding the scope of services that varies according to the remuneration system. Such as, in Nordic countries, asthma counselling service is the only remunerated service. In Australia, United Kingdom (UK) and New Zealand (NZ), many pharmacy services are being remunerated. Contrary to this, in countries like Malaysia, Hungary, Iran, Pakistan, Croatia, Jordan and Peru, pharmacists are not reimbursed for providing the professional pharmacy services [21, 22]. Another component involves the support of pharmacies network in order to fulfill the local needs and demands and improved access to medicines [20]. The last component depends upon a pharmacist’s ability to promote patient’s rational use of medicines through the delivery of cognitive pharmaceutical services, which are rewarded with little or no remuneration and reimbursement. Thus, in community pharmacy, the payment for the provision of pharmacy services is mostly confined to the dispensing of medicines or devices. In this scenario, a holistic approach is required whereby pharmacy professional organizations can formulate and negotiate with the government bodies for developing strategies to promote remuneration of pharmacy services. Besides, pharmacists also need to document and exhibit the value of pharmacy services including the provision of quality of such services [1, 21].

Pharmacy remuneration and payment for pharmacy services have been the key to promoting pharmacy practice and pharmacy public health activities. This has been evident in the countries where pharmacists’ remuneration has been already in place including Australia, UK, NZ and Canada. Payment for pharmacists has also been the basis to develop innovative pharmacy services. This has also led to an increase in global pharmacy practice literature. Moreover, the impact and value of evidence-based research about remunerated services may guide the policymakers and stakeholders to approve or disapprove a service based on its cost, value, and impact on the healthcare system.

Different pharmacy reimbursement models employed in various parts of the world are discussed below.

### Pharmacy reimbursement models

#### Fee-for-service (FFS)

The fee-for-service (FFS) means that healthcare providers are compensated according to the quantity of the services they render. The model encourages the pharmacists to actively provide the patient care services and allocates a fixed amount of fees for the pharmacists’ professional services, as well as covers the ingredient costs of the medicines [23]. Moreover, the model of services provides as much care as possible, regardless of the fact, if the service is necessary or otherwise [24].

Many disadvantages have been observed due to the standard incentives. A flat cost ignores the qualities of the professional services provided as they can get an incentive when a service is rendered. In order to generate more income, pharmacists may even have the potential to deliver more services than that of patients actually required [25]. Moreover, pharmacists may ask their patients to fill their prescriptions as quickly and as many as they can. On the contrary, if a pharmacist spends more time counselling a patient or urging them to postpone getting a prescription filled when it seems like they should still have enough medication, this will put the pharmacist at a financial disadvantage [23]. Additionally, patients may have a chance to overuse the medicine when the pharmacists dispense the medications more than the patients’ needs, in order to maximize their own profits. Also, pharmacists receive delayed reimbursement due to the complex and lengthy claiming process. Another disadvantage is for the pharmacists, who actively participate in prescription product selection and tracking patient drug profiles are not reimbursed at all under the FFS procedures [25].

The countries which apply the FFS are Australia, Canada, Belgium, Germany, Japan, the Republic of Korea, and Switzerland [26, 27].

#### Capitation

Capitation is a method of payment for health services, in which a fixed amount is prospectively paid to the health care provider, irrespective of the nature or quantity of services provided for each patient, registered with the provider [28]. In a community pharmacy, the capitation system pays the pharmacy, which was chosen by the payers. For instance, in the United States (US), capitation is provided to the pharmacies for their services to the Medicaid-eligible person on the first of the month. Instead of the number of prescriptions filled, a pharmacist is reimbursed based on the number of Medicaid-eligible patients on his list. Therefore, to retain those customers on the lists, pharmacists may improve the quality of their patients care services and reduce the costs of the prescriptions [29]. In order to save the medication cost, physicians on capitation payment models tend to administer fewer medications to patients with chronic illness [29]. Pharmacies receiving the cash payments at the beginning of each month, capitation model may limit the financial problem of the pharmacies by improving the continuity of cash flow. There is also a decreased workload for the community-based pharmacist practitioners as the processing and filling out claim papers would not be necessary in most cases [25].

The countries which employed capitation are the US, Thailand, Denmark, Indonesia, Netherlands, Portugal, and Ghana.

### Blended funding models

The single-base funding model’s shortcomings are addressed by the hybrid model known as blended funding model [23]. In a blended funding model, payment for pharmacy services provided by pharmacist is calculated and remunerated by both the government and private payers [30].

Several countries that practiced the blended funding models are Australia, New Zealand, China, and Canada [30]. In New Zealand, the blended system comprises capitation, patient co-payments, and targeted FFS. Similarly, the Diabetes Management Incentives have been enforced in Canada, as a blended funding model which comprises both the capitation and FFS model [30]. Table 1 below presents a comparison between different funding models.

**Table 1:** Comparisons between FFS, capitation, and blended funding models

Models	FFS	Capitation	Blended funding models
Definition	Healthcare providers are compensated by a fixed number of incentives based on the quantity of the services they provide	Healthcare providers receive payments at the beginning of each month according to the number of patients assigned to them	A hybrid model, mostly fee-for-services are integrated with capitation models
Advantages	Healthcare providers are keen to provide as many patient care services as possible	- The quality of services and cost-effectiveness of the treatment are improved - Pharmacy operators are less likely to have financial problems	Address the single-base funding model’s shortcomings
Disadvantages	- Healthcare providers may ignore the quality of the patients care services - Pharmacists may ask the patients to fill their prescriptions even if they still have enough medicines, which could lead to medicine over-usage - Pharmacists receive delayed incentives due to complex and lengthy claiming processes	- Less patients able to receive professional health services from pharmacists - Physicians may prescribe less medications to the patients to reduce the cost of treatment	–

A comprehensive overview of the reimbursement models in different countries is described below.

### Australia

The healthcare system of Australia is regulated by Medicare, which is a universal public health insurance [31]. In Australia, the Federal government and Pharmacy Guild of Australia signs an agreement every 5 years to remunerate pharmacy services and the remuneration is allocated to the community pharmacy for providing their services such as medication management and adherence. The various professionals services provided under this agreement are MedsCheck, Staged Supply and Home Medicines Reviews [32]. Availability of funding plays a key role towards practice change in this scenario. Thus to seek the benefits of such services over a long-term period, it requires the uncovering of the challenges in providing and evaluating the cost and associated benefits to the healthcare system [15].

Community pharmacy is also remunerated a fee of $44.86 (AUD $66.53)^1^ per patient for MedsCheck and $67.28 (AUD $99.79) for diabetes MedsCheck [33]. Similarly, for staged supply, a pharmacy can claim $5.47 (AUD $8.12) per patient as fee for first staged supply, and $2.78 (AUD $4.12) per patient for subsequent supply during the same week [31]. For home medicine reviews, a pharmacy and pharmacists are capped for 30 home medicine reviews per month. For initial review, a fee of $150.20 (AUD $222.77) will be claimed and for first and second follow-up, a service fee of $75.10 (AUD $111.39) and $37.56 (AUD $55.70) will be claimed, respectively [34].

The cost of the medicine to the pharmacist is approved as price to pharmacist (P2P) in an Australian community pharmacy. As of January 2021, according to the Pharmaceutical Benefits Scheme (PBS), drug administration, handing, and infrastructure (AHI) price to pharmacist ranges from $4.30 per maximum quantity supplied which is valued at less than $67.42 (AUD $100). Similarly, drug dispensing fee for the ready prepared medicine is $5.25 (AUD $7.78) per dispensed item, extemporaneously prepared medicine is $6.62 (AUD $9.82) per item and for dangerous drug the dispensing fee is $3.25 (AUD $4.82) per item [35]. For dose administering aids, a pharmacist is remunerated with $67.42 (AUD $100) twice a year to see whether the patient is managing well with dose administration aids or not [36].

### Canada

Medicare is the universal health insurance, funded by the federal government of Canada. But province-based governments are responsible to provide the primary health services and thus each territory or province provide their own universal insurance programs and therefore, differ in their community pharmacy remuneration [37]. In Quebec, when patients purchase medicines for that month, they pay a monthly set fee of $18.85, termed as deductible. Also, the patient will pay co-insurance, which is 34.50% of the price of the prescription after the payment of deductible. The payment from the pocket of the patient is termed as the patient’s contribution. The government then pays the difference between the prescription price and patient’s contribution [38]. The medicine coverage is limited to population in need, and a reduced co-payment fee is charged for prescription filling [37]. Community pharmacists in Alberta receive a dispending fee of $ 12.15 and for compounded prescription, up to $18.45 are remunerated [39]. Similarly, the advanced medicine review service is remunerated in three Canadian provinces. In Alberta, prescription renewal or extension, dose change receives $20 per assessment and in Saskatchewan it receives $6. For minor aliments assessment services, the remuneration fee in Quebec is $16, while $18 for Saskatchewan [40]. A community pharmacist in Alberta receives a fee of $14.92 (CAD 20) for pharmacist-administered vaccination or other injected medicines [41].

As the competition for market sharing is high, community pharmacies offer competitive fees, which range from $7 to $12 and sometimes it is as low as $2 only. In all Canadian provinces, a community pharmacist charges about $4.48–$14.99 (CAD 6-CAD 20.10) for prescription adaptation service, which is referred as the ability of a pharmacist to autonomously adapt an existing prescription. Interestingly, in Alberta, a pharmacist is remunerated with $14.92 (CAD 20) for non-dispensing service, which is based on the pharmacist’s professional judgment for not to dispense a prescription [40]. Besides, some immunizations services are reviewed and covered by the government. For example, Ontario-based patients, who are taking two or three medicines, MedsChecks service is provided to them by the government and the remuneration fee of $60 is paid to the community pharmacist, who can get $150 if he provides the review at patient’s home [37, 42, 43].

Further details on Canadian pharmacy services are given below in Table 2.

**Table 2:** Community pharmacy services remuneration in different states of Canada

State	Year	Service	Fee
Alberta [44]	2012	Comprehensive Annual Care Plan (CACP)	$74.60 (CAD 100) or $93.25 (CAD 125) if pharmacist has Additional Prescribing Authorization (APA)
British Columbia	2011	Medication Review (Standard) Refusal to fill	$44.76 (CAD 60) Two times dispensing fee
Newfoundland and Labrador (NL)		Medication Review (20–30 min) Refusal to fill Medication management	$39.17 (CAD 52.50) $16.26 (CAD 21.80) $8.13 (CAD 10.90)
Ontario	2007	MedsCheck MedsCheck for diabetes MedsCheck at home MedsCheck Follow-Up	$44.76 (CAD 60) $55.95 (CAD 75) $111.90 (CAD 150) $18.65 (CAD 25)
Saskatchewan	2012	Medication assessment Refusal to dispense Minor ailments program Adaptation	$44.76 (CAD 60) 1.5 times dispensing fee $13.43 (CAD 18) $4.48 (CAD 6)

### England

In England, the community pharmacies are contracted and commissioned under the national Community Pharmacy Contractual Framework (CPCF). The CPCF is responsible for the quality assurance of the services. There are 11,500 community pharmacies under the contract of National Health Services (NHS) in England. About 40 percent of the pharmacies are run by pharmacy contractors such as chain pharmacies and rest of the 60 percent is being operated by contractors such as large corporate pharmacy chains [45].

Under the NHS contract, pharmacies are responsible for providing three types of services, including essential, advanced, and enhanced services. The funding of community pharmacy is a complex mixture of income streams. It is a combination of remuneration and reimbursement from National Health Service (NHS) as part of their contract and many pharmacies receive additional income from both NHS and non-NHS sources, including fee per activity, payment for advanced and enhanced services, payment for commissioned services, retained margin, pharmacy quality and access scheme [45].

NHS England is responsible for paying fees and allowances to the pharmacies, which include single activity fee, item fee. This can be further divided into two categories, which include, payment for essential services and payment for advanced services [46]. The single activity fee for every dispensed item or device is $1.60 (127p) per item. A pharmacy can claim such additional fees which include dispensing of controlled drugs, dispensing of imported items or unlicensed specials and measuring and fitting hosiery items. An expensive drug valued above $125.70 (£100) can be charged as 2% of the total cost for an expensive prescription fee.

Since 2005, the out of hours’ service fee has been removed from the national NHS agreement and now it is commissioned by the local NHS team. Moreover, since 15th February 2021, the discharge medicines service (DMS) has been included in the essential services category, which can be cumulatively claimed as $44 (£35). If the DMS is provided in parts, the payment at stage one will be $15.08 (£12), $13.83 (£11) at stage two and at stage three, $15.08 (£12) can be claimed [47].

For advanced services, a pharmacy contractor receives a payment between $25.14–$35.20 (£20-£28) for each new medicine, depending upon number of patients, who receive the service in a month [37]. Similarly, the fee for conducting an appliance use review at pharmacy is $35.20 (£28) and at patient’s home is $67.88 (£54). Pharmacies in England also receive $12.04 (£9.58) per administered flu vaccine [48]. Besides for each community pharmacist consultation, $17.60 (£14) can be claimed [48]. Table 3 presents pharmacy services and their respective payments in England based on different programs.

**Table 3:** Payment of different community pharmacy services in England

Year	Program	Service	Fee
2005 [49]	Minor Ailments Scheme	Minor ailments consultation	$4.68–$10.93
2008 [49]	Medication Use Reviews	Medication use review	$42.16

### France

There are no chain pharmacies or online pharmacies in France, except the foreign online pharmacies, from where people can get their medicines. The Ministry of Social Security is responsible for setting and regulating the price of reimbursable medicines. The reimbursement rate of medicines is based on the severity of disease and effectiveness of medicine [22]. In France, the fixed dispensing fee of $1.10 (€1.02) per item is paid for reimbursed medicines, and $0.55 (€0.51) is paid on the dispensing of prescriptions having five or more medicines [37].

### Germany

Germany is the most populous country in the European Union with a population of 83.3 million [50, 51]. Mandatory health insurance is the key feature of German healthcare system and about 90% population get coverage from statutory health insurance. While the remaining 10% get health coverage through private insurance or special schemes [52]. As of June 2022, there are 18,256 community pharmacies in Germany and approximately 53,300 pharmacists are working in the community setting [50].

In 2004, the pharmacy fee was set at $8.73 (Euro 8.10) per prescription-only pharmaceutical, which slightly increased to $9.00 (Euro 8.35) in 2013 [53]. Previously there were no remuneration for community pharmacy services provided by the pharmacies, however after years of extensive research and negotiations, the “Law on Strengthening Local Community Pharmacies” was finally adopted by the German Federal Parliament. According to which all healthcare insurance companies are bound to pay about $161 million (150 million Euro) per year for the provision of community pharmacy services to the pharmacies, other than normal reimbursement incurred for prescribing and counselling the prescribed drugs [50, 51].

Since June 2022, there are five community pharmacy services, which are already approved to be billed by community pharmacies in Germany [50]. A pharmacist can provide one medication review for the patient, who is taking at least five systemic or inhaled drugs for long-term use [54]. The total duration of this medication review is on an average 80 min and is compensated at $97.01 including value-added tax (VAT) (EUR 90 + VAT). For medication review, related to follow-up for oral anticancer drugs or immunosuppressant post transplantation, the remuneration fee of $97.01 (EUR 90 + VAT) is charged for first medication review, while for second review, within two to six months of first medication review, the service is charged at $18.92 (EUR 17.55 + VAT) [50, 51]. Similarly for high blood pressure control in hypertension, in case of addition of new antihypertensive medicine, the pharmacy can charge $12.07 (each EUR 11.20 + VAT). If a patient receives a new inhalation device or switch to a new one, the service can be invoiced at $ 21.56 (EUR 20 + VAT) [50, 51].

### Indonesia

Community pharmacies are responsible for providing pharmaceuticals as well as the services for the patients, enrolled under the network of the primary care providers. In this system, pharmacies are reimbursed for pharmaceuticals through the pre-established electronic catalogue system of pricing. For medicine dispensing fee, the fee for individual patient is negotiated with network based on capitation payment. A network receives capitation payment of value less than $1 per patient, which is ideally considered to be distributed to both the network and pharmacy [55, 56].

On the other hand, a pharmacist receives a salary and other incentives from the government. The incentives include the dispensing fee, income from capitation payment, which is less than $0.50 per patient and incentives related to the delivery of healthcare campaign [55].

### Ireland

The healthcare system of Ireland is managed by the Government and the Department of Health and Children. The community pharmacists are remunerated for providing medicines and services to the Irish community under community drug schemes by Health Service Executive. Community pharmacists are paid a dispensing fee of $3.76–$5.38 (€3.50–€5), which is based on the volume of dispensed item under General Medical Services Scheme. When a patient is dispensed with a medicine under Long-Term Illness, a dispensing fee of $3.76–$5.38 (€3.50–€5) with an additional 20% mark-up is paid to community pharmacist. Moreover, a pharmacist in Ireland also receives a non-dispensing service fee of $3.52 (€3.27). In order to provide vaccination services to patients above 65 or having seasonal influenza, Irish government pays a fee of $16.13 (€15) to the community pharmacists [41].

### Japan

Japanese healthcare system is highly regulated by the government and healthcare is provided through universal public health insurance system available for all Japanese citizens and non-citizens [57].

Community pharmacists in Japan make most of their income through dispensing fee, which is paid by the health insurance program. Dispensing fees vary according to the prescription dispensed in a month and delivery of service thus dividing the dispensing fee into the type of service and size of pharmacy. Japanese community pharmacists receive a dispensing fee of $1.27–$3.49 (AUD $1.89–5.17) as well as $3.23–$4.25 (AUD $4.79–6.30) is paid for the provision of medicine and patient counselling [58].

### Malaysia

The Malaysian healthcare system is a two-tiered system, consisting of both public and private sectors. The public sector is heavily funded by the government and patients’ co-payments cover 3% of the cost of services. Malaysia adopted pharmacy services more than half a century ago, and since then the community pharmacists are trying to provide services beyond dispensing [59]. These services include but are not limited to the provision of medication management review, chronic disease management, smoking, and weight management. Currently, there is no central agreement in place for pharmacists’ remuneration by the government. The pharmacists are providing these services voluntarily [60]. A study by Shafie and Hassali (2010) has revealed that 67% of the surveyed individuals were willing to pay to pay for the dispensing services by pharmacist and the median amount was found as $2.86 [61].

### New Zealand

Twenty different district health boards in NZ are responsible for the allocation of funding to various health services in each territory [57]. In NZ, a patient is not registered to a particular pharmacy, but the patients are registered with specific general physicians working in a medical center. These medical centers work as private business while charging the fee as per the consultation and services provided to the registered patients [62]. In such a system, a patient may receive his prescription from one or more pharmacies and request for the pharmacy services from multiple pharmacies. As per the NZ Community Pharmacy Services Agreement, pharmacies are getting funding from one budget for the traditional pharmacy services, while other professional pharmacy services including handling and supply are funded through another budget [63]. A pharmacist is paid with the dispensing fee of $0.61 (NZ $1.00). Besides, up to 3 items, an initial dispensing fee of $3.06 (NZ $5.00) is charged and for repeat item $1.84 (NZ $3.00) per repeat item is charged [37, 64].

### Switzerland

The Council of Europe, in 1993 stated that the community pharmacies should be remunerated for the cognitive pharmaceutical services [65]. Thereafter, since 2001, a service-based remuneration system of community pharmacy has been established and is being remunerated by the health insurance [66]. The Swiss Pharmaceutical Association has published standards for pharmaceutical counselling and counselling provided during medicine dispensing is remunerated as drug check and deliver check [57, 66]. Six distinct services are eligible for remuneration, such as pharmacist fixed fee for advice, patient fixed fee for follow-up and record maintenance. Besides, emergency fixed fee to cover extra charges during emergency, night or weekend, directly observed treatment fixed fee, adherence fixed fee and generic substitution fixed fee also remunerated in Swiss community pharmacies [65]. In 2010, Polymedications Check service was introduced for patients who were having more than four medicines for over a period of 3 months and the remuneration fee was settled at $37.30 [49, 67].

### The Netherlands

The healthcare system is managed by the government and supplemented by the health insurance coverage provided by private insurance companies [68]. Community pharmacists are not remunerated for providing pharmacy services, except dispensing. A pharmacy will charge a fee of $7.53 (€7) for a prescription and the amount will be doubled, if it is dispensed for the first time [69]. For filling a prescription, the cost of prescription depends on drug price and pharmacy service charges, already set between health insurer and pharmacist. Moreover, the services charges by the pharmacist could be raised if the prescription is filled at weekends or during evenings [70].

Apart from this, community pharmacists receive a flat fee that covers all provided services by pharmacists. Thus, there is no incentive for pharmacists to provide quality or extra pharmacy services to patients, hence low rate of private consultations by community pharmacy can be seen in the Dutch community pharmacies [21].

### The United States

In the United States, different states have different models of remuneration to the pharmacy services based on the established regulations, the result of which is variability in reimbursement and compensated services. A multistate review by Nguyen et al. in 2020 has found that in California, pharmacists receive 85% of the scheduled fee equivalent to the physicians, and payments are not directly transferred to the pharmacist, but to the pharmacy. Similarly, in Alaska, the services are covered through payor regulation, however, pharmacists are not enrolled to insurance credentialing portals, thus they cannot provide the bills. Contrary to this, in New Mexico and California only specific pharmacies or pharmacists are allowed to bill. However, in Oregon state, the pharmacists need to contract and credential with each insurer, while insurers do not provide the payment [71]. Likewise, different states have different programs coverage, for example in the case of pharmacist-provided medication therapy management (MTM) services, 11 states (Michigan, Ohio, Virginia, Delaware, Missouri, Indiana, Minnesota, Kansas, North Dakota, Wisconsin and Tennessee) had Medicaid programs covering pharmacist-provided MTM services to some extent [72]. Table 4 below represents a summary of community pharmacy services in different parts of the US.

**Table 4:** Summary of community pharmacy services reimbursement across the US

State	Year	Service	Fee
California [49, 73]	2008	Comprehensive medication review Patient compliance consultation Patient education and monitoring	$76.70 $20.45 $10.23
California [49]	2007	Comprehensive medication review Patient compliance consultation Patient education and monitoring	$51.13 $20.45 $10.23
Colorado [49]	2007	Medication review	Face to face: $76.70 Telephone: $51.13
Florida [44]	2004	Comprehensive medication review Patient education and monitoring	$51.13 $10.23
Iowa [49]	1999	Comprehensive medication review Patient compliance consultation Patient education and monitoring	$51.73 $20.45 $10.23
Iowa [49]	2004	Comprehensive medication review Patient compliance consultation Patient education and monitoring	$51.50 $20.45 $10.23
Louisiana [49]	2011	Diabetes self-management training	$50.31 for 30 min/patient, $13.53 for 30 min/ person for group education
Michigan [49]	2010	Comprehensive medication review Patient compliance consultation Patient education and monitoring	$76.70 $20.45 $10.23
New York [49]	2010	Medication therapy management	For initial consultation: $35.79, For follow-up: $25.57
Texas [74, 75]		Diabetes medication management	For initial visit: $107.38 For follow-up: $56.25
West Virginia [49]	2010	Diabetes assessment	For initial assessment: $51.13 For Follow-up assessments $20.45 per 15 min

### Australian reimbursement system for community pharmacy: a case study

From ensuring access to essential medicines and promoting the delivery of quality pharmaceutical services to the population, the reimbursement system for community pharmacies plays a crucial role in the Australian healthcare system. Community pharmacies serve as key healthcare providers, bridging the gap between patients and healthcare professionals, and their sustainability relies heavily on an effective reimbursement system. Community pharmacies in Australia operate within a unique framework that balances the interests of healthcare providers, government agencies, and patients. The reimbursement system plays a critical role in maintaining this delicate equilibrium, ensuring pharmacies are adequately compensated for the services they provide while ensuring affordable access to medicines for patients [76].

In Australia, the community pharmacies provide two types of services based on different funding bodies. For example, medication management and medication adherence programs and trial programs are funded by the Sixth Community Pharmacy Agreement (6CPA) [77].

For medication adherence programs, incentives are divided on a pro rata basis among the participating pharmacies. Such as for staged supply of medicines, a community pharmacy may claim up to $5.47 (AUD 8.12) per patient for first staged supply and $2.78(AUD 4.12) for a subsequent supply, for about 15 patients per month. The claim for clinical interventions is based on the number of interventions per pharmacy for a specific period and is subject to a cap of 3.5% of prescription volume for each pharmacy [78]. Under Medication Management Programs, the community pharmacy is remunerated a fee of $ 67.28 (AUD 99.79) for Diabetes MedsCheck and $44.86 (AUD 66.53) per patient for MedsCheck [79]. Similarly, for the initial home medicine review, a fee of $ 150.20 (AUD 222.77) per patient may be claimed and for the first and second follow-up, an additional $75.10 (AUD 111.39) and $ 37.56 (AUD 55.70) can be claimed, respectively. Besides that, there are services, listed as unremunerated services or those paid by the patients, which include vaccination services [80].

The 6CPA has allocated up to $1.26 billion in funding for evidence-based, patient-focused professional pharmacy programs and services, which comprises $613 million for the continuation of a number of programs and services from Fifth Community Pharmacy Agreement (5CPA), $50 million for the pharmacy trial program; and up to $600 million for new and expanded community pharmacy programs [81]. The 6CPA is committed to analyze the Community Pharmacy Programs from the previous 5CPA. This assessment process supports a consistent approach to informing investment that delivers the greatest benefit to consumers. The review of Community Pharmacy Programs includes: Quality Use of Medicine Evaluation Report including staged supply evaluation, Clinical Intervention, Home Medicines Review, Dose Administration Aid, Residential Medication Management Review and MedsCheck and Diabetes MedsCheck [82].

Under the new Seventh Community Pharmacy Agreement (7CPA), the dispensing remuneration guarantee is introduced for the first time, and there will be an annual increment on the average remuneration per PBS prescription. Moreover, for the co-payment prescriptions, there is an increment of $763.251 million (AUD1.132 billion) in dispensing remuneration including a 9% increase in the dispensing fee of co-payment prescriptions to increase to $7.74 from 1 July 2020 [83].

Additionally, the Australian government has recently announced funding for community pharmacies to support more rural and regional communities. In this regard, more than $100 million are allocated for regional, rural, and remote pharmacies to better support their communities through an expansion to a critical regional pharmacy program, whereby previously $16 million per year were allocated under the previous 6CPA to now $21 million. Moreover, it is announced that over 400 additional pharmacies will be eligible for the Regional Pharmacy Maintenance Allowance (RPMA) program. Under this expansion, each eligible pharmacies will receive between $3000 to $51,328 per year, depending on their remoteness classification and PBS prescription volumes [84].

## Conclusion

Globally there is a need to understand that pharmacists’ roles and pharmacy services can only be improved if there is a payment for these services. The reimbursement systems for community pharmacies vary significantly across different countries, reflecting the diverse healthcare systems and policies worldwide. Countries like England, France, Germany, Ireland, and Switzerland have well-established reimbursement systems that recognize and compensate community pharmacies for the services they provide. On the other hand, countries like Japan and New Zealand primarily rely on dispensing fees as the main source of remuneration for community pharmacies. With significant variability in reimbursement models and services covered across different states, the United States presents a unique case. In Australia, the reimbursement system for community pharmacies presents a balance between fair compensation for pharmacies and affordable access to medications for patients. Moreover, the recent expansion of funding for regional and rural pharmacies highlights the Australian government’s commitment to supporting pharmacy services in underserved areas. Overall, the establishment of effective and fair reimbursement systems for community pharmacies is essential for the sustainability and growth of pharmacy services worldwide. It encourages pharmacists to expand their roles beyond dispensing and actively engage in patient care, ultimately contributing towards a better healthcare system.

## Data Availability

Not applicable.

## Footnotes

1 All currencies are converted according to the currency rates published on 10 June 2023.
